# The dynamics of dominance: open questions, challenges and solutions

**DOI:** 10.1098/rstb.2020.0445

**Published:** 2022-02-28

**Authors:** Eli D. Strauss, Daizaburo Shizuka

**Affiliations:** ^1^ Department of Collective Behavior, Max Planck Institute of Animal Behavior, Konstanz, Germany; ^2^ Centre for the Advanced Study of Collective Behaviour, University of Konstanz, Konstanz, Germany; ^3^ School of Biological Sciences, University of Nebraska Lincoln, Lincoln, NE, USA; ^4^ BEACON Center for the Study of Evolution in Action, Michigan State University, Lansing, MI, USA

**Keywords:** rank changes, social instability, social status, life history, transitivity, aggression network

## Abstract

Although social hierarchies are recognized as dynamic systems, they are typically treated as static entities for practical reasons. Here, we ask what we can learn from a dynamical view of dominance, and provide a research agenda for the next decades. We identify five broad questions at the individual, dyadic and group levels, exploring the causes and consequences of individual changes in rank, the dynamics underlying dyadic dominance relationships, and the origins and impacts of social instability. Although challenges remain, we propose avenues for overcoming them. We suggest distinguishing between different types of social mobility to provide conceptual clarity about hierarchy dynamics at the individual level, and emphasize the need to explore how these dynamic processes produce dominance trajectories over individual lifespans and impact selection on status-seeking behaviour. At the dyadic level, there is scope for deeper exploration of decision-making processes leading to observed interactions, and how stable but malleable relationships emerge from these interactions. Across scales, model systems where rank is manipulable will be extremely useful for testing hypotheses about dominance dynamics. Long-term individual-based studies will also be critical for understanding the impact of rare events, and for interrogating dynamics that unfold over lifetimes and generations.

This article is part of the theme issue ‘The centennial of the pecking order: current state and future prospects for the study of dominance hierarchies’.

## 1. Introduction

**Figure 1.**
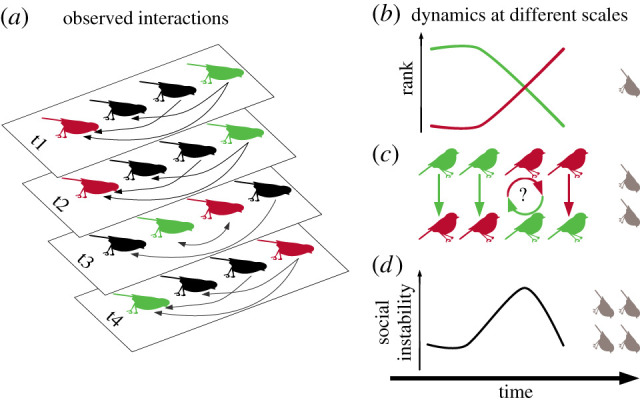
(*a*) Dominance hierarchies are inferred from observed agonistic interactions, depicted as a network sampled over four time periods (t1–t4; individual identity indicated for two individuals by colour). Arrows point from winners to losers, and the bidirectional arrow indicates cases where two individuals are each observed defeating the other. Dynamics within hierarchies occur at three scales (*b*–*d*, scale symbolized by pale birds on the right). (*b*) *Individuals* change position in the hierarchy. Here the two shaded individuals show opposite changes in rank over the study. (*c*) Dominance relationships within *dyads* change over time. Here, the two shaded individuals have a stable dominance relationship that reverses over the course of the study. In time-point t3, the birds have an uncertain dominance relationship. (*d*) Social instability reflects dynamics at the *group* level. (Online version in colour.)

Dominance is one of the most widely studied social behaviours, but is typically studied using a static approach in which agonistic interactions are tabulated and used to infer individual ‘rank’ in the dominance hierarchy [1–3]. These dominance ranks are then compared with other covariates of interest to understand causes and consequences of position in the dominance hierarchy in social systems [4]. Although the traditional static approach has produced valuable insight into the role of dominance in social systems, it side-steps challenges associated with the *dynamics* of dominance, i.e. changes in dominance hierarchies over time. As a result, many gaps remain in our understanding of how and why dominance hierarchies change over time and what impacts these changes have for of animal societies. Here, we highlight these gaps, discuss the challenges to addressing them, and suggest solutions to these problems and promising avenues for future research (table 1). Specifically, we examine research questions about dynamics of dominance occurring at three scales—individuals, dyads and groups (figure 1). Targeting these gaps in future research will provide an integrative understanding of how dominance operates dynamically to structure societies at multiple scales.

**Table 1.** A research agenda for the dynamics of dominance.

open questions	challenges	solutions
**individual level**		
how and why do individuals change position in the dominance hierarchy?	lack of conceptual clarity about rank dynamics at individual level	conceptual distinction between *inter-* and *intragenerational mobility* and *active* and *passive mobility*
	accurately measuring social mobility	account for uncertainty in rank measurement when identifying changes
		determine appropriate time-scale at which to assess social mobility
how do dominance trajectories across life produce fitness trajectories and impact selection on status-seeking behaviour?	it is difficult to study processes occurring at lifetime scale	long-term individual-based studies
		theoretical models integrating behaviour and dominance trajectories
**dyadic level**		
when and why do dyads engage in contests?	requires data that go beyond direct interactions—e.g. initiation, avoidance, long-distance signals, behavioural state, etc.	develop methods for studying the lack of interactions
		account for opportunity to interact
		distinguish the roles of dominant and subordinate individuals in driving interaction rates
how do dominance relationships form and dissolve?	requires high-resolution interaction data	captive systems with the capacity for high-resolution data collection (e.g. automated tracking)
	lack of theoretical framework to guide empirical studies	development and testing of interaction-to-relationship models and cognitive models of dominance relationships
**group level**		
what are the causes and consequences of social instability?	lack of conceptual clarity about social instability	conceptual distinction between *membership, rank* and *aggression network instability*
	accurately measuring instability	research into appropriate time-scale at which to measure instability
		account for uncertainty in rank measurement when identifying hierarchical instability
	rare but extreme instability can have high impact but be difficult to study	long-term studies that capture naturally occurring extreme instability
		experimental manipulation of social instability

## 2. Individual level

### (a) How and why do individuals change position in the dominance hierarchy?

Social rank has important consequences for individuals, impacting stress physiology, social relationships, longevity, immune function and reproductive success [5–8]. For most species, it is unclear what causes individuals to change position in the dominance hierarchy, or conversely, how dominants may preserve their status [9,10]. It is important to understand the causes and consequences of rank changes [11], both to understand potential selection on status-seeking behaviour [12–14], and because rank changes can shed light on the forces involved in determining social rank in the first place [15,16]. However, progress in understanding the dynamics of dominance hierarchies is hampered by lack of a clearly defined concept of ‘rank change’. The literature is plagued with redundant and ambiguous terminology such as rank change [17,18], rank reversal [19,20], revolutionary coalition [21], dominance turnover [22,23], social mobility [24–26] and power trajectories [27]. The proliferation of related terms reflects the complexity of the concept—i.e. that position in the dominance hierarchy can change in multiple ways. Thus, there is a need for multiple rank-change concepts and clear distinctions between them.

We borrow concepts from the study of social mobility in humans to delineate categories of how rank changes can occur. Social mobility can occur between generations—*intergenerational mobility*—or within generations—*intragenerational mobility* [28]. Intergenerational mobility measures the extent to which parental dominance rank predicts offspring dominance, whereas intragenerational mobility describes movements of individuals in the hierarchy over their lifetimes. There are two types of inter- and intragenerational mobility that arise via different processes [29]: *active mobility*, which involves a reversal of a previously held rank relationship and *passive mobility*, which is a change in rank that occurs without any reordering of the hierarchy. Passive mobility results from demographic processes like births/deaths and immigration/emigration—for example, if the highest-ranked individual dies and no active intragenerational mobility occurs, all remaining individuals improve their ranks by one position through passive intragenerational mobility [30,31]. Drivers of active mobility are less well-understood, but this type of mobility could result from changes in covariates that influence rank (e.g. increase in social support [12,15,32] or resource holding potential [22]), by stochastic outcomes that are reinforced (e.g. by winner/loser effects [33]), or feedbacks between multiple processes [34,35].

Recent work on hierarchy dynamics in spotted hyenas (*Crocuta crocuta*) illustrates the various forms of social mobility. In this system, social rank is highly predictable based on the rank of the mother, in a process termed ‘maternal-rank inheritance’, which is also observed in many old world monkeys [36–39]. Such systems represent an extreme version of restricted intergenerational mobility, because a female's rank is strongly correlated with the rank of her mother. Intragenerational mobility occurs through active and passive processes in this system. Active intragenerational mobility occurs when lower-ranking females overtake their higher-ranked groupmates through coalitionary support [15]. Passive intragenerational mobility owing to reproduction drives increasing differences among individuals and lineages over time [15]. This example demonstrates how distinguishing among these different types of social mobility will help to bring conceptual clarity to research into hierarchy dynamics and will reveal diverse drivers and impacts of mobility.

Methodological groundwork exists for inferring patterns of social mobility, but more work in this area is needed. Mobility can be measured in absolute units (e.g. increase/decrease in number of individuals dominated) or relative to other members of society (e.g. increase/decrease in rank standardized for group size) [40,41]. Contrasts in the causes and consequences of relative and absolute mobility can reflect biological differences in competitive landscapes; absolute mobility is expected to be more important when the resources over which animals compete are density dependent, whereas relative mobility is expected to be more important when these resources are density-independent [42]. Many methods exist for inferring a rank order from a sample of animal contests [43,44], and numerous studies have evaluated the efficacy of these methods at finding rank orders [44–46], but very little work has evaluated the efficacy of these methods for inferring changes in rank over time. Consequently, applying these existing methods to the study of social mobility will require some refinements. First, if social mobility is rare, then noise in calculations of social rank will make it difficult to distinguish true mobility events from false identification of rank changes [29]. Thus, the study of social mobility requires the development of approaches that accurately estimate social mobility and account for uncertainty (box 1). Additionally, more work should focus on measuring intergenerational mobility. To measure intergenerational mobility, researchers can use parent–offspring correlations between rank, as is often done in economics. An alternative approach is to compare observed offspring rank to a rank based on a reference model where offspring win and lose interactions with equal probability as their parents [59]; this approach may be less biased by differences between parents and offspring in observation time or interaction rate. Finally, more work needs to address how to decompose mobility into active and passive components. Techniques have been advanced for decomposing changes in ordinal rank (e.g. rank 1, 2, …, *n*) into passive and active mobility [29], but this method does not work for cardinal ratings (e.g. David's scores, Elo-rating), which are sometimes preferable (e.g. when measuring hierarchy steepness; [47,60,61]). In summary, a fruitful path forward is to continue refining methods for inferring hierarchy dynamics at the individual level.

Box 1. **Methodological challenges in inferring hierarchy dynamics.**

A few studies have made progress towards improving the efficacy of ranking methods for identifying mobility, but considerable work remains. Approaches that determine ranks based on discrete subsets of the data and infer changes by comparing these rank orders overestimate the true amount of mobility [29]. This issue can be alleviated by using an ‘updating’ process to rank individuals in each study period based on prior ranks informed by newly collected data. This updating approach is implemented by default in the Elo-rating and Glicko-rating methods [47–51], but can also be incorporated into other commonly used types of ranking methods such as David's scores or matrix reordering [29]. An issue with approaches that update scores after each encounter (e.g. Elo-rating and Glicko) is that they require some data to be allocated to an initial ‘burn-in’ period during which hierarchy position and dynamics are discarded as part of a process of statistical convergence, leading to lost data. This problem can be exacerbated when there is a high degree of demographic turnover and initial data for new individuals are reserved for burn-in [52]. Solutions for this problem include using prior information to help place new individuals [29,52] or using statistics to estimate starting scores of new individuals based on the outcomes of early interactions [53,54].

A crucial methodological decision when identifying social mobility is to determine the time period over which potential dynamics are assessed. The more frequently potential changes are assessed, the more potential changes can be found. For instance, assessing an individual's change monthly over a year can lead to the identification of 11 changes in position, whereas measuring mobility daily over the same period could potentially identify 364. Accordingly, sampling for dynamics more frequently leads to the identification of more changes [29]. There are dangers to assessing potential changes both too frequently or too infrequently—if changes are assessed too rarely, real changes can be missed or misinterpreted (i.e. false negatives) [47], while assessing changes too frequently can lead to inference that is overly sensitive to uncertainty in an animal's relationships (i.e. false positives). If only a few individuals or interactions are sampled during the periods over which mobility is assessed, this will lead to an overestimation of the number of changes and an underestimation of the rate of change (i.e. rank instability; see Group level section). Data-splitting approaches can be used to assess the timescale over which a rank order is predictive of future interaction outcomes [55], providing a guide for the appropriate time-scale over which to assess potential hierarchy dynamics. Finally, we recommend a sanity check for a correspondence between the particulars of a given study (e.g. question of interest, study organism) and the time-scale over which hierarchy dynamics are assessed. For instance, assessing hierarchy dynamics over very short time-scales is appropriate for studies focused on fine-scale patterns in the emergence of hierarchical social structure in small groups of short-lived animals [56]. By contrast, assessing hierarchy dynamics over longer time-scales is more appropriate for studies of the fitness consequences of dominance trajectories in large groups of long-lived species, where some individuals may only interact infrequently and the outcome of interest (e.g. reproductive success) operates over long time-scales [15]. In this sense, we advise against a default paradigm of assessing dynamics daily or after every interaction, as is currently typically done with the Elo-rating method.

The last challenge for measuring social mobility is identifying and accounting for uncertainty. There is a pressing need to expand methods for detecting social mobility to account for uncertainties in rank orders. Otherwise, measurement error can lead to the overestimation of social mobility and lead the noise of spurious social mobility to swamp the signal of true social mobility. This is particularly challenging because it is difficult to distinguish *measurement uncertainty* in rank order—arising from sampling bias, observer error and missing data—from *biological uncertainty* in rank relationships among individuals [57]. In fact, because active intragenerational mobility by definition involves changing dominance relationships, biological uncertainty in rank orders is expected to increase during periods of active mobility. Therefore, a crucial step is to develop methods for measuring and interpreting uncertainty in estimates of social mobility. The Glicko-rating, randomized Elo-rating and percolation and conductance (PERC) methods incorporate approaches for quantifying uncertainty around inferred dominance ranks or scores [45,49,58], but no study has yet used these uncertainty estimates when inferring hierarchy dynamics.

### (b) How do dominance trajectories across life produce fitness trajectories and impact selection on status-seeking behaviour?

Dominance rank is often linked to fitness [8], but we know relatively little about the temporal dynamics of these effects. Effects of rank could be ephemeral, with each instance of rank change causing corresponding changes in rank-related outcomes [11,31,62], or they could be persistent and manifest even after individuals undergo social mobility [63]. Moreover, the way in which individuals move through the hierarchy over the course of their lifetime can moderate short-term influences between rank and fitness [8,10,64]. For instance, the costs of dominance status acquisition can offset the benefits of high rank [65–67], making it necessary for individuals to hold high status for sufficient time to gain a net benefit. Furthermore, individuals could all show similar trajectories over life—in such a case, subordinates may appear to be paying a fitness cost by being subordinate, when instead they will eventually enjoy dominant status, and in fact all individuals may experience relatively equal lifetime fitness. The dynamics of rank across development (e.g. being raised by humans is associated with reduced dominance in juvenile greylag geese (*Anser anser*) [68]) and life-history stages (e.g. dispersal in spotted hyenas [69]) add further complexity to the ways that dynamic rank links to fitness.

Critically, in addition to modulating short-term associations between rank and fitness, dominance trajectories can reflect selection on status-seeking behaviour or influence the stability of social systems. For instance, some have suggested that an on-average tendency to improve in social status over the life course is critical for maintaining persistent groups [41]. Theoretical work suggests that if subordinates can achieve high status by queuing, this relaxes selection on status-seeking behaviour and could lead subordinates to be more tolerant of despotism by dominants [64]. Subordinate individuals with similar rank may vary in status-seeking behaviours (e.g. information collecting, prospecting, challenging dominants) that later influence their trajectory in social status [35,70–72]. In summary, to truly understand the influence of rank on fitness and the evolution of status-seeking behaviour, it is necessary to examine dominance trajectories over individuals' lifetime to understand how fitness outcomes vary as a function of rank and mobility over the life course. Here, theoretical models of optimal strategies under different dominance trajectory regimes [64] and long-term individual-based studies will be particularly valuable.

This life-course approach of dominance trajectories also opens an opportunity to take a life-history view of status-seeking behaviour. From this perspective, how individuals invest in status-seeking behaviour across a lifetime will depend on a combination of the fitness consequences of status, the longevity of such effects and the probable mechanisms of rank change (i.e. intra- versus intergeneration mobility, active versus passive mobility) [64,71]. For example, in systems where rank and fitness are highly correlated, and upward social mobility is largely passive, selection may favour life-history strategies that increase longevity to maximize the chances of attaining high rank by persisting in the queue. Conversely, in systems where active mobility predominates, selection may favour early investment in growth in order to maximize the probability of displacing dominants. Such integration of social dynamics and life-history theory will contribute to an emerging perspective on life history of social behaviour [73–75]. In total, viewing dominance rank as a trajectory that unfolds over the life course will reveal typical patterns of dominance trajectories, potential alternative strategies to maximizing fitness in hierarchical societies, and the role of social mobility in the evolution of status-seeking (or status-preserving, e.g. [76]) behaviour.

## 3. Dyadic level

### (a) How do dominance relationships form and dissolve?

A century ago, Schjelderup-Ebbe [1] presented a simplistic verbal model of how dominance relationships form and change, stating of a contest between hens A and B: ‘If B wins she will become the despot, possibly forever but in any case for the time being’ [1, p. 36]. Over a century of research on dominance, considerable progress has been made in understanding how the outcomes of interactions influence individual behaviour and physiology, but the dynamics of dyadic relationships are less well-understood. What processes lead some dominance relationships to form and persist, whereas others change, and still others are never formed?

A major insight from the last century of dominance research is that dominance relationships are influenced by the social context in which they operate—that is, dyadic dominance relationships are not determined in a vacuum, but are instead influenced by other dyadic relationships [77–79]. Dyads in newly formed groups tend to form dominance relationships producing transitive triads, demonstrating how the formation of relationships plays a causal role in shaping the formation of other relationships within the group [78,80,81]. A survey of dominance hierarchy structure across broad taxonomic groups confirms that this tendency towards transitive triads is a reliable feature of dominance hierarchies [82]. Most recently, work in chickens, cichlids and mice tracking all interactions among small newly formed groups provides an in-depth look into how dominance hierarchies emerge and persist after formation, showing that even after establishment, shifting dominance relationships still tend to change from one transitive network to another [56]. These results suggest that dominance hierarchies are best thought of as existing in a state of ‘dynamic stability,’ where dyadic relationships and individual positions in the hierarchy change but the overall transitive structural feature of the hierarchies remains constant. This impressive literature reveals why some dominance relationships are more likely to form than others, but we still do not know what processes produce the dynamics in dyadic relationships that give rise to this dynamic stability.

Individual and dyadic interaction history are processes that can contribute to the dynamics of dyadic dominance relationships. Theoretical and empirical work has demonstrated that dominance interactions lead to winner and loser effects, where the winners (losers) of interactions perceive themselves as more (less) able to win contests, and thus increase (decrease) their probability of winning subsequent interactions [33,83–85]. These winner- and loser-effects operate in addition to intrinsic differences in individual competitive ability to affect individual rank [86], but it is less clear how such effects impact dyadic relationships. Insofar as dominance relationships result from the combination of interactions [87,88], these effects of prior interaction experience are expected to influence dominance relationship formation [33]. However, in many species, individuals recognize groupmates, so dominance relationships formed between pairs of individuals are impacted by their specific dyadic interaction history [16,89–91]. When two individuals interact, the status of their dominance relationship is probed, reinforced or altered [87]. For unfamiliar individuals, repeated interactions quickly lead to the establishment of a dominance relationship, which is characterized by an overall reduction in aggression [92]. Repeated interactions can also lead to a change in how dominance relationships are assessed. For instance, in golden-crowned sparrows (*Zonotrichia atricapilla*), experimental enhancement of head plumage to signal higher dominance influenced dominance relationships among strangers but not among familiar flockmates, suggesting a move from reliance on status signals to recognition-based mechanisms of dominance relationship assessment [93]. In established relationships, additional interactions typically reinforce the existing dominance relationship, but can sometimes counter it and lead to its reversal. Individual-level changes such as winner/loser effects or changes in competitive ability play a role in the dynamics of these relationships, but are insufficient to fully explain these dyadic phenomena. Future work can shed new light on the evolution of dominance by exploring how individuals integrate information from prior interactions with specific opponents to form stable yet dynamic dyadic relationships.

Specifically, a productive way to deepen understanding of how dominance relationships form and dissolve requires the development of *interaction-to-relationship* models of how repeated interactions with particular opponents are integrated to form relationships [94]. These models should be able to reproduce typical patterns of dominance relationships, where established relationships form, remain stable, but can also change to a new stable state after new interactions—that is, relationships that once formed remain stable ‘possibly forever, but in any case for the time being.’ Feedback loops between interaction outcomes and their determinants (e.g. body size, resource holding potential) suggest mechanisms by which stable dominance relationships might be pushed over a tipping point [34,95]. Interaction-to-relationship models need to consider: (i) potential time dependency in the influence of interactions on relationship status [96], (ii) effects of social context on the dyadic dominance relationship [78,79], and (iii) underlying cognitive models by which individuals understand their relation to their groupmates.

Empirical studies point to some alternative plausible cognitive models underlying dominance relationships. Individuals may track group consensus about position in the dominance hierarchy [97], track the aggression received by group members and use it to infer position in the hierarchy [98], monitor aggression network structure using transitive inference [98], remember their specific relationship with other members of the group [99], attend to signals reflecting competitive ability [100] or some combination of these models. These models make predictions about how dominance relationships might change under different perturbations, such as the removal of the dominant individual, changes in physical condition, social mobility among other group-members or stochastic outcomes of interactions that do not align with the dominance relationship. These cognitive models also imply differences in access to third-party information and other social information about the ranks of groupmates [101,102]. Theoretical models and agent-based simulations [103] present a promising venue to establish where models make different predictions about the dynamics of dyadic relationships. Empirically testing many of these models may require complete or nearly complete interaction data, so these tests are best suited for captive systems that support high-resolution data collection [92], potentially aided by automated data collection [104].

### (b) When and why do dyads interact?

Why do some dyads compete more than others? We know that in many species, attributes of dyads—for instance, kinship, size similarity or sex-homophily—influence the frequency of agonistic interactions within dyads [105,106]. Rank differences between individuals also shape interactions [76,107], for instance leading to increased likelihood of escalation of interactions among closely ranked individuals [108]. Recently, aggregated data on dominance interactions across a broad array of species has examined the occurrence of multiple rank-difference-based patterns of aggressive contests [101]. In the ‘downward heuristic’ pattern, dyads interact at random with respect to rank differences. By contrast, in the ‘bullying’ pattern, dyads with increasing rank differences are more likely to interact, and in the ‘close-competitors' pattern, dyads with increasing rank differences are less likely to interact [101]. This work suggests potential strategies determining when and why dyads choose to interact, inferred from these social dominance patterns. More work is needed to understand the processes that give rise to these patterns [107], how they change over time, and what they reveal about the dynamics of dyadic dominance relationships.

Interaction-to-relationship models (see previous section) are likely to make different predictions about the occurrence of these social dominance patterns. Newly formed groups of monk parakeets (*Myiopsitta monachus*) show unstructured aggression early after group-formation but quickly converge on the close-competitor pattern, indicating how these patterns may reflect the process of dominance relationship formation [98]. A promising future direction is to inquire how interaction strategies combine with different interaction-to-relationship models to influence the stability of dyadic relationships and overall hierarchical stability (see next section). Are certain strategies more effective at ensuring the stability of dyadic relationships? For instance, under some interaction-to-relationship models, bullying the lowest-ranked group member is predicted to reinforce dyadic dominance relationships broadly with other group members, whereas under other models it is predicted to only influence the dyadic relationship of the bully and her target. Addressing this question will reveal how dyadic interaction strategies influence dominance hierarchy dynamics across scales [109].

A challenge for understanding when and why dyads interact is that aggregated interaction data do not contain full information on the processes that influence dyadic interaction. These data only reflect interactions that occurred, but avoidance, long-distance signals and behavioural state can influence how dyads interact by eliminating interactions [105,110]. Furthermore, dyadic interactions could be driven by the behaviour of the dominant or the subordinate member of the dyad (e.g. a subordinate approaching a dominant who is feeding), but agency over the interaction is often assumed to belong to the dominant individual. A solution to these problems is to incorporate data on these other covariates into analysis of dyadic interaction rate. For instance, Dehnen *et al.* [107] account for spatial subgrouping when calculating their measures of the tendency for vulturine guineafowl (*Acryllium vulturinum*) dyads to interact, reflecting interaction decisions after accounting for the opportunity to interact. Incorporating data on the initiation of interactions (e.g. approaches) can reveal the extent to which dominant or subordinate individuals are influencing dyadic interaction rates.

## 4. Group level

### (a) What are the causes and consequences of social instability?

Schjelderup-Ebbe [1] hypothesized that dominance hierarchies serve to regulate conflict among group-members. A corollary to this hypothesis is that social instability—i.e. changes to a social group's dominance hierarchy—leads to increased conflict and its associated costs. Thus, an ongoing area of research is aimed at identifying periods of instability and determining the consequences of social instability for group members [111–113]. If instability is often not costly, this would challenge the idea that stable hierarchies arise as conflict regulatory adaptations [114]. Finally, there may be feedback between social instability and dominance-related traits, where competitive strategies differ in species with stable hierarchies compared to those with unstable hierarchies. For these reasons, to understand the role of dominance dynamics in animal societies, it is critical to explain the causes and consequences of social instability.

**Figure 2.**
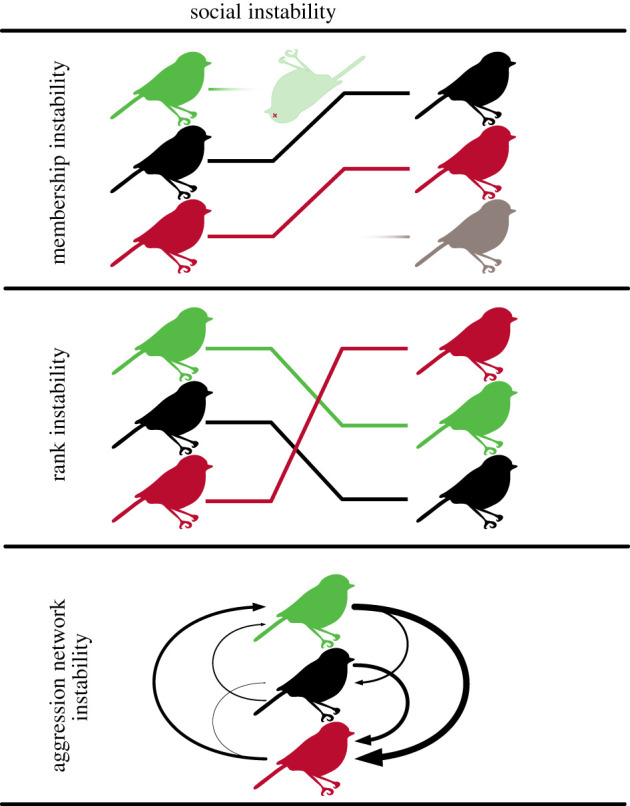
Three types of social instability. Membership instability results from demographic turnover. Rank instability results from rearrangements of the order of individuals within the social hierarchy. Aggression network instability results from a reduction in orderliness (e.g. transitivity, directional consistency) of the aggression network. (Online version in colour.)

A major challenge to the study of social instability is to agree on what it is, how to talk about it and how to measure it. In some studies, social instability is defined as a measure of changes in group composition [113,115,116], for instance owing to the loss or gain of many individuals or the occurrence of group fission. In other studies, instability is defined by rearrangements of the dominance hierarchy or by changes in individual-level dominance rating over time [47,52,117]. Instability is also sometimes defined a third way, as a reduction in orderliness of the aggression network. Here, instability is measured by an increase in intransitivity in dominance relationships [112], or by an increase in the frequency and inconsistency of dominance interactions [118]. Although thematically linked, these different types of instability do not necessarily arise from the same processes or have the same consequences. In order to properly understand sources of social instability and its impacts on animals, it is crucial to refine the concept to distinguish between these different patterns. We suggest distinguishing *membership instability*—caused by demographic turnover [75]—from *rank instability*, caused by changes in the ordering of individuals in the hierarchy. Finally, *aggression network instability* is defined by an increase in uncertainty and intransitivity in aggression networks [57] (figure 2).

Distinguishing among these types of instability is especially important because they can interact in important ways. Demographic turnover can have direct effects on dominance hierarchies by removing or adding individuals and their relationships with others in the group, but can also have indirect effects on other individuals [75]. Influx of new individuals can lead to rank instability—this is especially common in species with multi-male groups where males compete for dominance. For instance, during the mandrill (*Mandrillus sphinx*) mating season, an increased influx of males leads to increased intra-sexual competition, more active mobility among males and consequently higher rank instability, and higher levels of oxidative damage in high ranking males [111]. The loss of certain key individuals can also lead to rank instability [119,120] and aggression network instability [121], or even group collapse [122]. Membership instability, rank instability or aggression network instability may be more impactful if it occurs in the upper portion of the hierarchy [47,120]. Despite these avenues for interaction between types of social instability, it is also possible for each to occur independently of the others. Finally, in natural populations, extreme instability of these different types may occur rarely but have a large impact on animal societies [123], emphasizing the need to study these processes over long time-scales.

Methods exist for quantifying these different types of social instability, but again this is an area where there is room for improvement. To quantify membership instability, similarity metrics [124–126] can be used to assess differences in group composition between two time periods, even when group membership is not binary. Future work should aim to identify a metric that optionally weights measures of demographic turnover by the attributes (e.g. sex, rank) of individuals who join or leave the group.

Multiple approaches exist for quantifying rank instability. One approach is to calculate an index based on the amount of active mobility taking place from one study period to the next. The S index [47] measures hierarchical instability in this way, but it has some shortcomings—‘study periods' have a fixed length of 1 day, mobility among highly ranked individuals is weighted more heavily than others, and there is no way to account for measurement uncertainty. Future work should aim to extend this approach to assess instability over more biologically relevant time frames (box 1; [55]), incorporate measurement uncertainty [45], and optionally weight instability among all individuals equally. Aggression network instability can be measured from the aggression network itself, for instance as frequency of the occurrence of intransitive triads [82] or the amount of uncertainty in the network [58]. However, doing so relies on the assumption that intransitivity reflects instability rather than a stable but intransitive state [112,114], an assumption which has received some support [94] and some criticism [127] and will probably vary by species. It could be productive to break the network into components and measure features of those components separately. For instance, the Helmholtz–Hodge decomposition can be used to break an aggression network into the sum of a unique perfectly transitive network and a unique perfectly cyclical network—aggression network instability can then be measured as the cardinality of the cyclical graph [128]. This approach could also allow for independent study of cyclical and transitive elements of the aggression network.

## 5. Conclusion

Dominance hierarchies are enigmatically both stable and dynamic. As a repeated pattern of asymmetry in agonistic outcomes between individuals, the concept of dominance is founded upon some element of stability [88]. However, dominance relationships can also undergo rapid reversals, leading sometimes to dramatic changes in individual rank and group-level social instability. Nevertheless, even when relationships change, hierarchies gravitate towards the same underlying structural state of transitivity [56].

After a century of research on dominance hierarchies, we are still left with many questions to explore about how and why dominance hierarchies change over time, and what impact these changes have on animal societies. Hierarchy dynamics occur at three scales—individual, dyadic and group (figure 1)—and open questions remain about the dynamics of dominance occurring at each of these scales (table 1). We have known for some time that individual ranks change over time (e.g. as individuals grow and age), but conceptual clarity about the different forms of social mobility will aid us in making sense of how evolution has moulded social traits and status-seeking behaviour in the context of life history. One critical need is to extend methods for inferring dynamics at the individual and group scales. These methods need to account for measurement uncertainty, and guidelines are needed for determining the time-scale at which to assess hierarchy dynamics. Fortunately, these are already active areas of research [29,47,55,129]. At the dyadic level, more work is needed to understand when and with whom individuals choose to interact [101], and how these interactions are integrated to form a relationship [94]. Here, a combination of model development and studies in captive groups provide a promising avenue for insight through an iterative process of model testing and refinement. Captive groups where high-resolution interaction data can be collected are promising systems in which to test different interaction-to-relationship models [56,92]. Across scales, study systems where rank can be manipulated (e.g. [62]) will be extremely useful for conducting targeted experiments testing hypotheses about the causes and consequences of the dynamics of dominance. There is also room for work integrating studies of the dynamics of dominance with other forms of social power [130]. Finally, long-term individual-based studies will be essential for interrogating dynamics occurring at long time-scales and for studying the impact of rare events. We hope that this research agenda enables new insight into the dynamics of dominance and further extends the last century of productive research into this fundamental dimension of social organisms.

## Data accessibility

This article has no additional data.
